# Evaluation of automated techniques for extraction of circulating cell-free DNA for implementation in standardized high-throughput workflows

**DOI:** 10.1038/s41598-022-27216-5

**Published:** 2023-01-07

**Authors:** Sarah Lehle, Julius Emons, Carolin C. Hack, Felix Heindl, Alexander Hein, Caroline Preuß, Katharina Seitz, Anna L. Zahn, Matthias W. Beckmann, Peter A. Fasching, Matthias Ruebner, Hanna Huebner

**Affiliations:** Department of Gynecology and Obstetrics, Comprehensive Cancer Center Erlangen-EMN, Erlangen University Hospital, Friedrich-Alexander-Universität Erlangen-Nürnberg (FAU), Universitätsstrasse 21-23, 91054 Erlangen, Germany

**Keywords:** Breast cancer, Tumour biomarkers, Cancer, Biomarkers, Molecular medicine, Biological techniques, High-throughput screening, Isolation, separation and purification

## Abstract

Analysis of circulating cell-free DNA (ccfDNA) is a suitable tool for detecting somatic mutations for the purpose of making decisions on treatment, monitoring treatment response, and predicting survival. High-throughput techniques for ccfDNA extraction are essential to implementing ccfDNA testing in the clinical setting. We set out to compare two automated techniques with regard to hands-on time, ccfDNA output and integrity, and circulating mitochondrial DNA (mtDNA). CcfDNA was isolated using the EZ1&2 ccfDNA field test kit (EZ2 kit, QIAGEN) and the Maxwell RSC ccfDNA plasma kit (Maxwell kit, Promega). DNA was extracted from plasma of 30 breast cancer patients enrolled in the iMODE-B (#325_19B; 12.10.2020) study. Real-time PCR, fluorescence-based detection and automated electrophoresis were used to assess ccfDNA concentrations. The ccfDNA yield was significantly higher when extracted with the EZ2 kit. The EZ2 kit enabled the isolation of a higher proportion of short fragments and a lower proportion of long fragments, resulting in lower DNA integrity. Significantly lower mtDNA quantities were detected in the Maxwell eluate than in the EZ2 eluate. Thus, decisions on which extraction method to use should proceed on the basis of the required input for downstream applications, the anticipated fragment size and minimum hands-on time.

## Introduction

For many cancer entities, liquid biopsy has emerged as a source of novel, sensitive biomarkers for diagnostic and predictive purposes^1^. Liquid biopsies enable the quantification and evaluation of tumor components such as circulating tumor cells, tumor-specific antigens, and circulating tumor DNA (ctDNA)^1,2^. CtDNA is a fraction of the circulating cell-free DNA (ccfDNA) which is derived from tumor cells. CcfDNA can be released into the blood circulation after cell apoptosis or necrosis or through active release from, for example, tumor cells^3^. The analysis of ccfDNA isolated from the plasma of cancer patients has provided comprehensive, non-invasive insights into tumor-specific genetic and epigenetic alterations^4–6^. Research has found that, alongside certain genomic features (e.g. tumor-specific mutations), ccfDNA parameters such as quantity and integrity are suitable tools for the monitoring of disease progression, for providing guidance for managing treatment, or for use as a diagnostic biomarker^7–9^. One example of this work is the finding that post-surgery detection of ctDNA is a highly accurate biomarker for metastatic relapse after curative neoadjuvant treatment of breast cancer patients^10,11^. Further, the isolation and analysis of ccfDNA has recently gained importance due to its potential to disclose the occurrence of novel tumor-specific *PIK3CA* or estrogen receptor 1 mutations, which can lead to therapeutic resistance and are associated with worse outcomes^12–15^**.** Similarly, responses to immunotherapy have been linked to the presence of chromosomal instability, identified by analysis of ccfDNA using next-generation sequencing^16^. This research further underlines the high potential of ccfDNA for use in various clinical settings.

The quality of ccfDNA is a crucial matter for downstream applications. One major issue is contamination of ccfDNA with white blood cell DNA. The contaminant DNA can derive from leukocyte lysis during blood draw, blood transportation, or processing^17^. It is therefore important to critically evaluate ccfDNA samples prior to downstream analysis, by, for example, analyzing the fragment profile using specialized electrophoretic methods. The choice of appropriate blood collection tubes and rapid processing of the samples have been found to prevent leukocyte lysis^17^. High molecular weight DNA can negatively influence library yield and the sequencing quality of downstream analysis; this underlines the importance of evaluating fragment length distribution using a method such as automated electrophoresis.

Beside the quality of ccfDNA, DNA yield is a decisive limitation of downstream analysis. Most ccfDNA contains repetitive sequences such as short and long interspersed elements (SINE and LINE respectively)^9^. With a copy number of 1.4 × 10^6^ per genome, ALU elements are the most abundant sequences of the human genome; with a size of only approximately 300 nucleotides, they are classified among the SINE elements^17^. Quantification of ALU elements by quantitative real-time polymerase chain reaction (qPCR) is commonly used to determine ccfDNA concentrations and to assess the integrity of ccfDNA^9,18^. The integrity of ccfDNA is defined as the ratio of long to short or total ALU repeats^19–21^. The assumption here is that integrity represents the ratio of cancer cell-derived DNA to DNA derived from normal cells^19,22,23^. This is because ccfDNA released from naturally apoptotic cells consists of uniformly truncated small (< 200 bp) DNA fragments, while tumor cell necrosis generates fragments larger than 200 bp^22,24–26^.

Researchers have discussed the number of ALU fragment copies within the ccfDNA as a diagnostic and prognostic cancer biomarker; increased levels of ccfDNA integrity may be associated with the presence of breast cancer^9,27^. In line with these indications, additional evaluation of ccfDNA levels and ccfDNA integrity have been shown to significantly improve the sensitivity of a combined diagnostic test based on the circulating tumor markers carcinoembryonic antigen and the mucin-1 specific antigen CA15-3^28^. The clinical relevance of integrity makes its determination a standard procedure in the field of ctDNA. Interpretation of integrity needs to proceed in awareness of the bias caused by the method of isolation used^20,29^. This bias arises from differences in the abilities of different methods of isolation to preferentially isolate longer or shorter fragments^30^. Determining integrity is therefore of considerable interest in the comparison of new methods of isolation^20,29,30^. We note here that the term “integrity” does not reflect the quality of a sample per se, as it simply describes the composition of fragments.

Another novel biomarker representing a fraction of ccfDNA is circulating mitochondrial DNA (mtDNA)^31–36^. For example, total mtDNA levels in breast cancer patients may be associated with prognostic parameters such as histological grade, tumor stage, lymph node status, and hormone receptor status^37^. Further, the finding that baseline mtDNA copy numbers in patients with manifest breast cancer were significantly higher than copy numbers in cancer-free individuals indicates the diagnostic potential of mtDNA^38^. The analysis of breast cancer risk-associated SNPs in mtDNA may be suitable for incorporation into breast cancer prevention and screening programs^39,40^. Only 0.001% of the ccfDNA, however, can be mapped to the mitochondrial genome, and mtDNA levels in plasma are 180-fold lower than in fresh tissue samples^41^. These low levels of mtDNA are challenging for downstream assays such as whole genome sequencing and SNP genotyping; clinical applications therefore require sensitive and standardized ccfDNA isolation methods which result in a high quality and quantity of mtDNA.

With applications of ccfDNA multiplying, there is an emerging demand for high-throughput, standardized ccfDNA isolation technologies^42^. In this context, automated systems are crucial to providing reproducible and robust results and facilitating the integration of ccfDNA markers into clinical routines. The high standard of automation, fast throughput and low hands-on time achieved by magnetic bead-based isolation kits makes them the method of choice in clinical routines, preferred to silica membrane-based techniques. Despite general indications that silica membrane-based methods isolate higher yields of total ccfDNA and mutant ctDNA, some comparative studies have shown that the Maxwell kit isolates yields of ccfDNA equal to those isolated using silica membrane-based methods^30,44,45^. These studies observed that, with regard to ccfDNA quantity, the Maxwell kit produced yields equal to or even higher than those generated by the manual gold-standard kits QIAamp Circulating Nucleic Acid kit and QIAamp minElute ccfDNA mini kit (QIAGEN, Hilden, Germany)^29,30,43–45^. The Maxwell RSC ccfDNA plasma kit (Maxwell kit; Promega, Madison, WI, USA) is currently considered the gold standard for magnetic bead-based isolation^29,43^. CcfDNA extraction methods used in routine diagnostics have to be labor— and cost-efficient, and their ccfDNA yield needs to be of high quality and quantity and to enable robust results on ccfDNA integrity and sufficient mtDNA output. In light of these considerations, the aim of the present study was to compare the ccfDNA concentrations, integrity, and mtDNA yields attained by two fully automated magnetic bead-based methods for the isolation of ccfDNA from plasma samples.

## Materials and methods

### Patients

We recruited patients within the iMODE-B (Imaging and Molecular Detection of Breast Cancer) study, whose purpose is to identify molecular biomarkers within a high-risk collective (BI-RADS 4 and 5) at the time of breast cancer screening^46,47^. Patients were eligible for inclusion if they had a suspicious breast lesion for which a diagnostic biopsy was indicated. The analysis included samples from 30 participants with histologically confirmed breast cancer.

The study was conducted in accordance with the guidelines of the Declaration of Helsinki, and approved by the Ethics Committee of the Faculty of Medicine at Friedrich-Alexander-Universität Erlangen-Nürnberg (FAU) (#4514) (protocol code 325_19 B, date of approval: October 12, 2020). All participants gave their written informed consent before enrolling in the study. Only women aged 18 years or older were included. Clinical data were available for all 30 breast cancer patients (Table 1). The patients had a mean age of 62.4 years.

**Table 1:** Patient characteristics of breast cancer cases showing means and standard deviations (SD) for continuous characteristics and frequencies and percentages for categorical characteristics.

Clinical predictor	Cases (n = 30)
Age at blood collection [years; mean, SD]	62.4 (13.4)
BMI (kg/m^2^, median, IQR)	27 (6.5)
**Grading [n; %]**	
G1	10 (32)
G2	13 (43)
G3	7 (23)
Lymph node status [n; %]*	
N0	20 (83)
N1-3	4 (17)
Tumor stage [n; %]**	
T1	15 (52)
T2	12 (41)
T3	1 (3)
T4	1 (3)
**Breast cancer subtype [n; %]**	
TNBC	3 (10)
Luminal A	22 (73)
Luminal B	3 (10)
HER2	2 (7)

*BMI* Body mass index; *IQR* Interquartile range; *TNBC* Triple-negative breast cancer; *SD* standard deviation.

*data only available for 24 patients ** data only available for 29 patients.

### Sample collection and plasma preparation

The blood samples were collected in 10 ml Streck Cell-free DNA BCT blood collection tubes (Streck, La Vista, NE, USA) and centrifuged within two hours of blood draw. The literature describes Streck Cell-free DNA BCT blood collection tubes (Streck, La Vista, NE, USA) as suitable for ccfDNA isolation due to the preservative they contain, which prevents leukocyte lysis^17^. The blood samples were processed within 24 h of blood draw and samples with visible leukozyte lysis (red color of plasma) were excluded from further processing. Blood samples were centrifuged at 1600 × g for 10 min to separate lymphocytes from plasma. Subsequently, the supernatant was centrifuged for another 10 min at 16,000 × g to clear the plasma of remaining debris. The supernatant was stored in 1 ml fractions at 80 °C until use.

### ccfDNA extraction

We extracted two samples of ccfDNA from each study participant, using either 1) a demo version of the EZ2 Connect Instrument (EZ2 instrument, QIAGEN, Hilden, Germany) with the EZ1&EZ2 ccfDNA field test kit (EZ2 kit; QIAGEN, Hilden, Germany) or 2) the Maxwell RSC instrument (Maxwell instrument, Promega, Walldorf, Germany) with the Maxwell kit; we used each set of equipment in accordance with the manufacturer’s instructions (Table 2). Plasma input volume was 1 ml each. Due to the EZ2 instrument requiring a minimum volume of 2 ml, 1 ml of PBS was added to the plasma sample as recommended by the manufacturer’s protocol. The eluted ccfDNA was stored at − 20 °C until further downstream analysis. We avoided multiple cycles of freezing and thawing.

**Table 2:** Specifications of ccfDNA extraction kits used.

Kit	Maxwell RSC ccfDNA plasma kit (16)	EZ2 ccfDNA kit (24)
Manufacturer	Promega	QIAGEN
Method	Magnetic beads, automatic	Magnetic beads, automatic
Input volume (ml)	0.2–1.0 (in this project: 1.0)	2, 4 or 8 (in this project: 1.0)
Elution volume (µl)	50–70 (in this project: 70)	Default / not variable
Recovered volume (µl)	In this project: 65	75
Hands-on time (min)	10	45 (30*)
Incubation/centrifugation time (min)	–	–
Automated runtime (min)	70	36
Total runtime (min)	80	81 (66*)

* Calculated for 16 samples.

### Quantification of total ccfDNA

Total ccfDNA quantity was assessed using the QuantiFluor dsDNA System kit (Promega Corporation, Madison, WI, USA) and the Quantus fluorometer (Promega, Walldorf, Germany). We complemented these processes by using the Agilent TapeStation (Agilent Technologies, Santa Clara, CA, USA) and the Cell-free DNA ScreenTape assay (Agilent Genomic ScreenTape, Agilent Technologies) to assess ccfDNA concentrations and percentages of ccfDNA per total DNA yield. We predefined a ccfDNA region of 50–700 bp in order to differentiate between ccfDNA—including multimeric fragments—and high molecular weight DNA.

### Quantification of ALU copy numbers by quantitative real-time polymerase chain reaction

We measured ccfDNA fragments via a qPCR assay, targeting repetitive ALU elements as described previously^29^. In brief, one short (ALU60) and one long (ALU247) ALU fragment were amplified by qPCR. The primer annealing sites of the short fragment were located within the primer annealing sites of the long fragment. Consequently, the ALU60 primers amplified both the long and short fragments, representing the total amount of ccfDNA fragments longer than 60 bp, whereas the ALU247 primer set selectively amplified long fragments. For the short ALU fragment, a product of 60 bp was amplified with 5′-GGAGGCTGAGGCAGGAGAA-3′ as the forward and 5′-ATCTCGGCTCACTGCAACCT-3′ as the reverse primer^48^. For the 247 bp ALU product, the primer set was: forward 5′-GTGGCTCACGCCTGTAATC-3′; reverse: 5′-CAGGCTGGAGTGCAGTGG-3′^49^.

Numbers of ALU copies per ml plasma were calculated using a standard curve with serial dilutions of gBlocks Gene Fragments (10–0.01 ng/μl) (Integrated DNA Technologies, USA) as described previously^29^. We additionally calculated approximate concentrations of ccfDNA using a standard curve with serial dilutions of known germline DNA concentrations and quantification of ALU60 repeats.

CcfDNA integrity was calculated as the ratio between ALU247 and ALU60 copy numbers per μl ccfDNA determined by qPCR. In each case, the primer annealing sites of ALU60 were within the ALU247 annealing sites, resulting in DNA integrity of 1.0 when the template DNA is not truncated, and 0.0 when ccfDNA is completely truncated and thus solely contains fragments smaller than 247 bp.

The qPCR reaction contained 3.2 μl of the extracted ccfDNA and 9.3 μl master mix (SYBR Select Master Mix [applied biosystems], 0.2 μM forward and 0.2 μM reverse primer). The thermal cycling conditions were as follows: 50 °C for 2 min, 95 °C for 2 min, followed by 40 cycles of 95 °C for 15 s and 60 °C for 60 s. Each reaction was conducted in triplicate. Positive and negative controls were added to each qPCR run.

### Quantification of hmito copy numbers by quantitative real-time polymerase chain reaction

We established the amount of mtDNA by targeting a mtDNA-specific 65 bp site (hmito) within the template ccfDNA. The sequence for the primer set used was: forward 5′-CTTCTGGCCACAGCACTTAAAC-3′ and reverse 5′-GCTGGTGGTGTTAGGGTTCTTTGTTTTT-3′^50^. qPCR conditions were in accordance with the ALU–based qPCR. gBlocks Gene Fragments were used to assess the number of mtDNA copies per μl ccfDNA, as previously described^29^.

### Statistical considerations

We used Graph Pad Prism version 9.0.2 (GraphPad software, San Diego, CA, USA; https://www.graphpad.com/) for statistical analysis and for plotting of graphs. Parametric model assumptions were assessed using a quantile–quantile plot or a Shapiro–Wilk test. Mean values between two groups were compared using a paired Student´s t-test for normally distrbuted data and a Wilcoxon test for non-parametric analysis. Statistical relationships between two variables were assessed using Pearson’s correlation coefficient (r). All tests were two-sided and *p* values ≤ 0.05 were considered statistically significant.

## Results

### Automated ccfDNA isolation methods

CcfDNA from 1 ml plasma was extracted using the Maxwell kit (16) and the EZ2 kit (24). Both extraction methods use magnetic beads for ccfDNA purification (Table 2). The EZ2 instrument can handle undiluted plasma volumes ranging from 2.0 to 8.0 ml, while the maximum input volume for the Maxwell instrument is 1.0 ml (Table 2). The automated runtime and the total runtime for 16 samples were shorter for the EZ2 than for the Maxwell instrument (36 and 66 min vs. 70 min and 80 min respectively), while the hands-on time was considerably longer with the EZ2 (30 min vs. 10 min respectively; Table 2).

### CcfDNA yield

**Figure 1:**
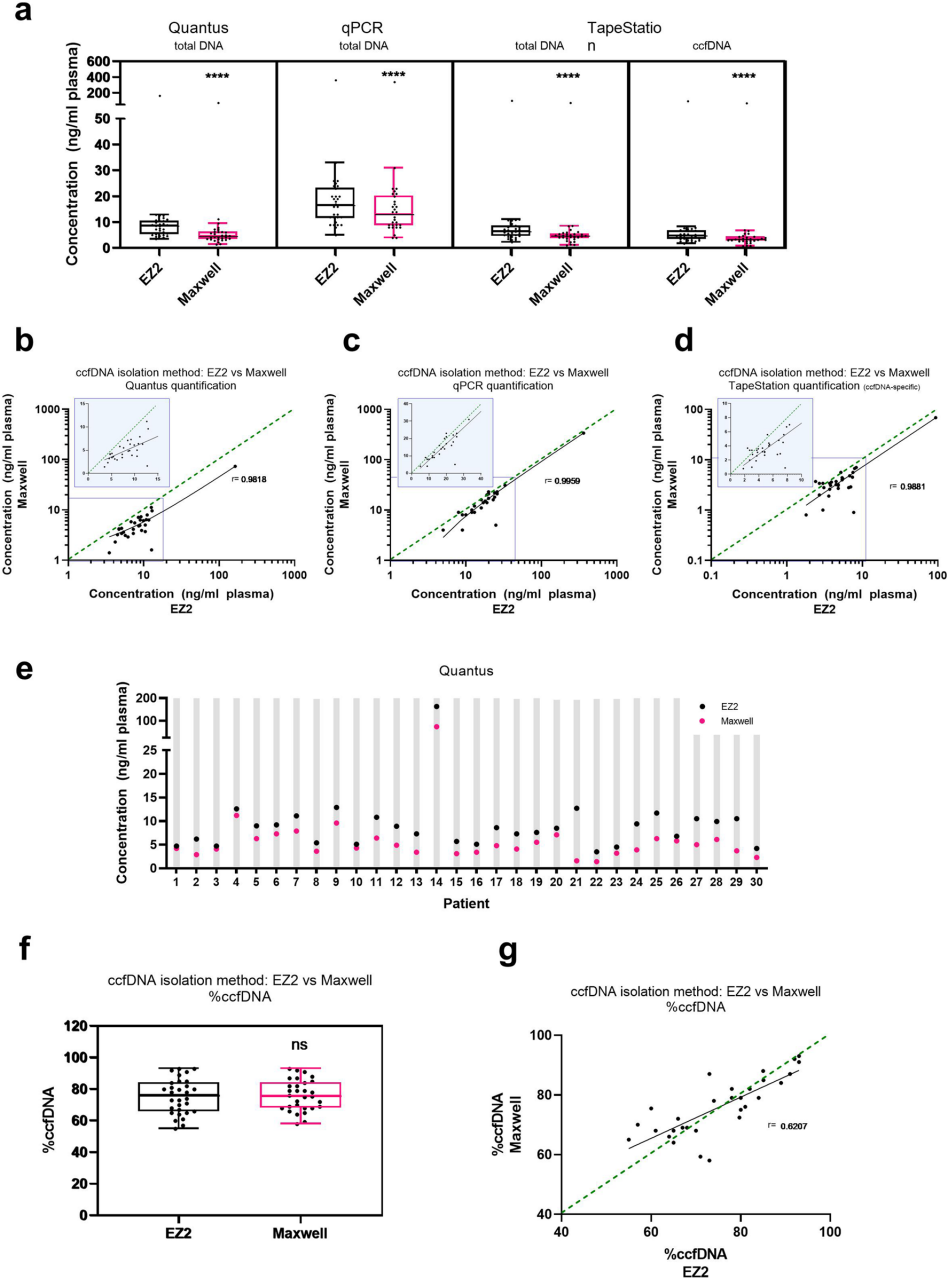
Concentration of isolated circulating cell-free DNA (ccfDNA). (**a**) CcfDNA was isolated from paired plasma samples using the EZ2 ccfDNA field test kit or the Maxwell RSC plasma ccfDNA kit. The concentrations (ng/ml plasma) were determined by Quantus fluorometer, quantitative real-time-polymerase chain reaction (qPCR), or TapeStation analysis. The central line in the box plot represents the median value and the whiskers are Tukey whiskers. Correlations of ccfDNA concentrations in EZ2 samples with ccfDNA concentration of Maxwell samples were evaluated for quantification by (**b**) Quantus fluorometer, (**c**) qPCR quantification, and (**d**) TapeStation. The green dashed line represents the bisector, and the solid black line the linear regression. The blue graph inserts represent magnifications of the marked areas of the original graph. (**e**) Concentrations of ccfDNA isolated by the EZ2 and Maxwell kits and quantified using Quantus fluorometer were presented for each patient. (**f**) The percentage of ccfDNA (%ccfDNA) was measured and calculated using TapeStation analysis. (**g**) Percentages of ccfDNA per samples isolated using the Maxwell kit were correlated with ccfDNA percentages of samples isolated using the EZ2 kit. The central line in the box plot represents the median value and the whiskers are Tukey whiskers. ****, *p* ≤ 0.0001. n = 30.

We quantified concentrations of total DNA isolated from cell-free plasma using fluorescence-based detection (Quantus fluorometer), qPCR, and automated electrophoresis (TapeStation). All quantification methods showed a significantly higher total DNA yield from samples extracted using the EZ2 kit than from those extracted via the Maxwell kit (*p* < 0.0001, *p* < 0.0001, *p* < 0.0001 and *p* < 0.0001 respectively) (Fig. 1a). The fluorescence-based method revealed a median DNA yield of 8.6 ng/ml plasma for samples isolated with the EZ2 kit and 4.6 ng/ml plasma for those isolated with the Maxwell kit (Fig. 1a). The DNA concentrations of EZ2 eluate correlated with the Maxwell concentrations regardless of the quantification method used (Fig. 1b–d). The angle of the correlation curve referring to the qPCR data is slightly steeper and closer to the bisecting line than the correlation curves of the fluorescence data and the elecrophoresis data. The EZ2 instrument obtained higher ccfDNA concentrations than the Maxwell instrument for all samples analyzed (Fig. 1e). The median difference of the amount of ccfDNA obtained by Maxwell versus EZ2 extraction was 2.9 ng per sample (Fig. 1e). The proportions of ccfDNA calculated in total extracted DNA did not differ between the isolations using the EZ2 and the Maxwell instrument (Fig. 1f). DNA electrophoretic profiles from three patients show the peak of the mononucleosome and a smaller peak representing the dinucleosome (Supplemental Fig. S1). The ccfDNA proportions of samples obtained with the EZ2 and Maxwell kits correlated significantly (r = 0.6207, *p* < 0.0001) (Fig. 1g).

### CcfDNA fragment length composition

**Figure 2:**
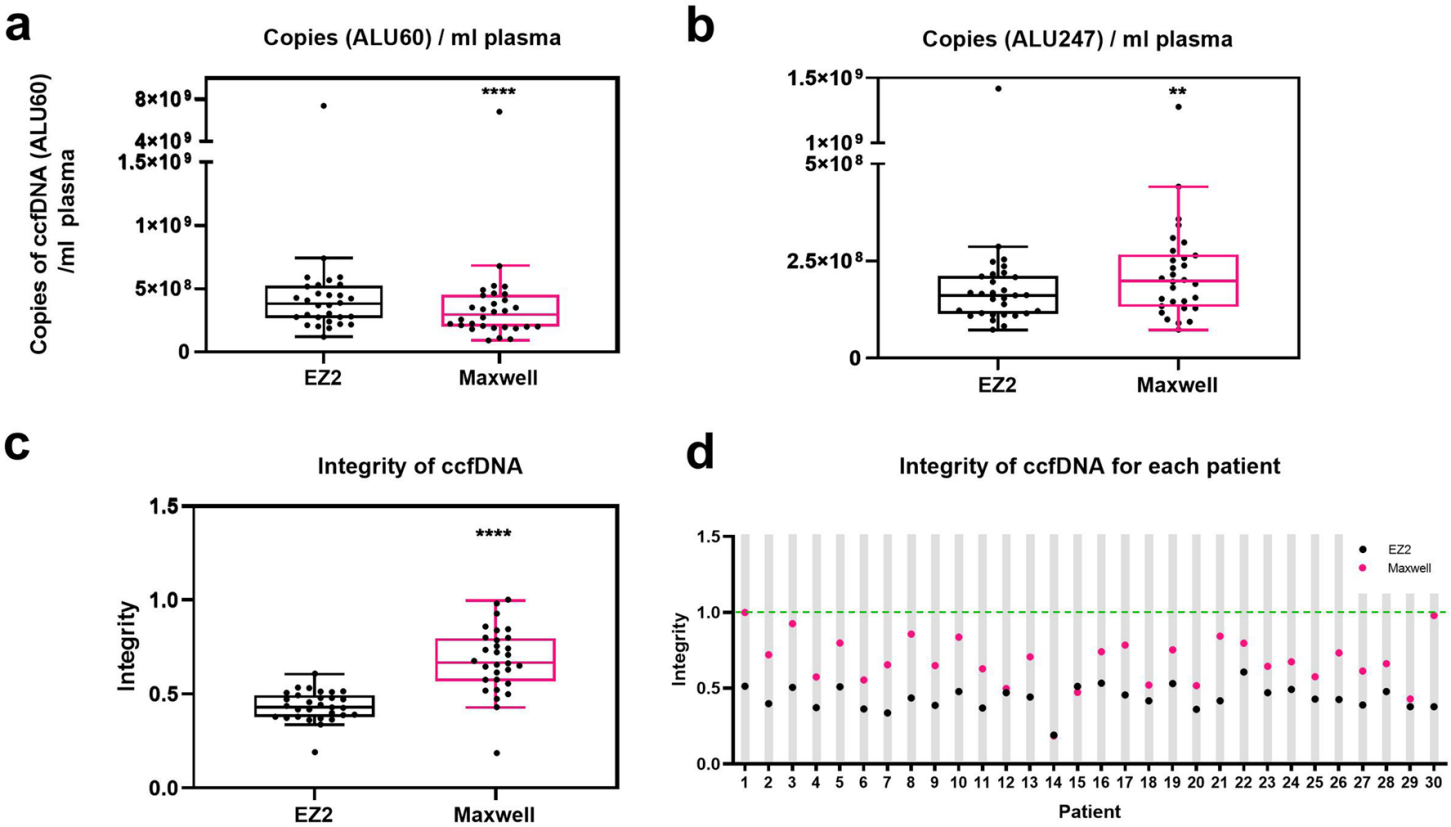
Integrity of circulating cell-free DNA (ccfDNA). Copies per ml plasma of short (**a**) (ALU60) and long (**b**) ALU fragments (ALU247) were quantified by quantitative real-time-polymerase chain reaction (qPCR) in EZ2 ccfDNA field test kit isolates and Maxwell RSC ccfDNA plasma kit isolates. (**c**) The integrity was presented as the ratio between ALU247 and ALU60 copy numbers. (**d**) Integrities of ccfDNA were presented per patient. The central line in the box plot represents the median value and the whiskers are Tukey whiskers. **, *p* = 0.004; ****, *p* ≤ 0.0001. n = 30.

Significantly higher ALU60 copy numbers were measured in the EZ2 kit extracts than in those isolated by the Maxwell kit (median copy numbers of 3.8 × 10^8^ and 2.9 × 10^8^ per ml plasma respectively; *p* < 0.0001) (Fig. 2a). Conversely, ccfDNA isolated using the Maxwell kit contained a median ALU247 copy number of 2.0 × 10^8^ per ml plasma, compared to 1.6 × 10^8^ copies in the EZ2 kit samples (*p* = 0.004) (Fig. 2b). In addition, the ratio of longer fragment size to total fragment size (integrity) of EZ2 and Maxwell samples differed significantly (*p* < 0.0001) (Fig. 2c). With a median difference of 0.22, the integrity of Maxwell extracts was higher than that of those obtained by EZ2, for all samples analyzed (Fig. 2d).

### Circulating mtDNA yield

**Figure 3:**
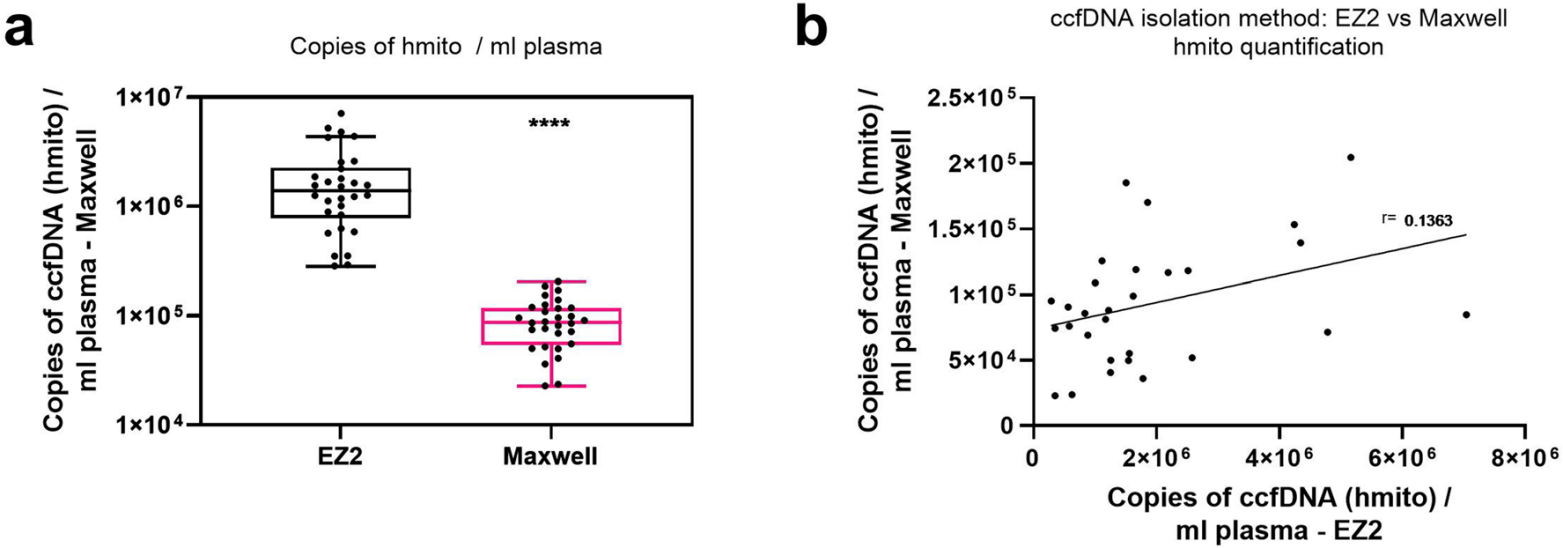
Copies of circulating mitochondrial DNA fragments (hmito). (**a**) Copies of hmito fragments per ml plasma were quantified by quantitative real-time polymerase chain reaction (qPCR) in samples isolated using the EZ2 ccfDNA field test kit or the Maxwell RSC ccfDNA plasma kit. (**b**) Copies of hmito isolated using the EZ2 ccfDNA field test kit were correlated with the copy numbers of hmito fragments in samples isolated with the Maxwell RSC ccfDNA plasma kit. The solid black line indicates the linear regression observed. The central line in the box plot represents the median value and the whiskers are Tukey whiskers. ****, *p* ≤ 0.0001. n = 30.

Copy numbers of mtDNA were quantified by qPCR, targeting a specific hmito sequence. The mean number of mtDNA copies was significantly higher when extracted with the EZ2 kit (*p* < 0.0001) (Fig. 3a). The median copy numbers of hmito in the EZ2 ccfDNA samples were 15.9-fold higher than those in the Maxwell samples (Fig. 3a). The copy numbers per ml plasma of samples extracted with the Maxwell instrument and the EZ2 kit did not correlate (r = 0.1363) (Fig. 3b).

## Discussion

The automated isolation of ccfDNA is crucial to applications within large-scale projects. Automated extraction of ccfDNA could help to minimize human error, enable high-throughput downstream assays and implement standardized procedures for diagnostic tests. Both the EZ2 (QIAGEN) and the Maxwell (Promega) instruments reduce hands-on time for ccfDNA isolation compared to manual kits^29,51^. For the QIAamp MinElute ccfDNA kit, which is a manual-only protocol, a total experiment time of 50 min was reported for the processing of 16 samples^29^. Contrastingly, the hands-on times of the Maxwell kit and the EZ2 kit were 10 min and 30 min respectively. Notwithstanding the relatively long hands-on time observed with the EZ2 kit, the total runtime was lower (66 min) than that of the Maxwell instrument (80 min). The reason for the EZ2 instrument’s hands-on time being 20 min longer than that of the Maxwell relates to the necessity of handling multiple tubes, each of which requires placing in the appropriate positions in the EZ2 tip rack and unscrewing separately by hand. However, the EZ2 kit and EZ2 instrument were a field test set; the equipment is still in the development process. It may therefore be the case that changes in the setting will be taken into account during finalization of the instrument for commercial use. Unlike the Maxwell instrument, the EZ2 instrument offers the option of processing samples in the tubes they were stored in. However, these tubes need to match the instrument and to fit into the EZ2 rack. Not all biobanks may have tubes in use that match this system; 2D matrix tubes, for example, are in common use, but are not compatible with the EZ2 system. Alongside a rapid and reproducible extraction process, sufficient ccfDNA output is an important factor in enabling in-depth analysis of phenomena such as alterations within ctDNA. The low copy numbers of mutant alleles behind an extensive background of wild-type DNA present a technical challenge to downstream analytical platforms^52^. When mutation type and position are known, it is possible to perform very sensitive methods such as droplet digital PCR (ddPCR); otherwise, the screening of whole DNA fragments requires next-generation sequencing (NGS) methods^52^. For such downstream applications, a ccfDNA input of between 1 and 25 ng is necessary^53^. The EZ2 kit achieved a median output of 8.6 ng from 1 ml plasma used, while the Maxwell kit yielded approximately 4.6 ng. Both of these yields are within the range of necessary input; however, for in-depth analysis using, for example, NGS methods, input of more than 1 ml of plasma would be necessary, particularly when using the Maxwell kit. qPCR-based quantification returned a median ccfDNA yield of 17.0 ng/ml plasma for EZ2 kit isolates and 13.0 ng/ml plasma for Maxwell eluates. These ccfDNA plasma concentrations were in the lower range of ccfDNA concentrations reported by others for early breast cancer (12–52 ng/ml)^54–56^. In these studies, qPCR-based quantification was a frequent method of choice^57^. More than 50% of the cohort of patients included in our comparison of the EZ2 and Maxwell kits had a tumor stage of T1; an early tumor stage is generally associated with lower levels of ccfDNA. Further, 83% of the patients were lymph node-negative (Table 1). Research has found that the concentration of ccfDNA in plasma correlates with the size of the tumor and that lymph node-positive patients show higher levels of ccfDNA^9,55^. Several research groups have also reported comparably low ccfDNA plasma levels^22,56,58^. In a non-triple negative cohort consisting mainly of stage II breast cancer patients, the median ccfDNA concentration was 13.22 ng/ml^22,58^, which is in line with the yield achieved by EZ2 and Maxwell isolation. Interestingly, we detected lower ccfDNA concentrations when measured with fluorescence-based quantification compared to qPCR quantification. It was shown earlier that fluorescence-based DNA values were dependent on salt concentrations and decrease non-proportionally to the dilution ratio^59^. In addition, fluorescence dyes, such as PicoGreen, specifically bind to double-stranded DNA (dsDNA)^59^. CcfDNA, however, consists of both dsDNA and single-stranded DNA (ssDNA) with a higher proportion of ssDNA^60^. Thus, quantification of ccfDNA using fluorescence dyes—in particular such kits designed for dsDNA (e.g. QuantiFluor dsDNA System kit)—might underestimate the ccfDNA concentration.

The EZ2 and Maxwell kits were both bead-based isolation methods. This technique enables a high standard of automation and is thus attractive for a variety of clinical applications. The high level of automation in bead-based methods does, however, come with the drawback of lower plasma ccfDNA yields than are attainable with manual silica-based methods. There is controversy in the literature in this area, with some researchers arguing that bead-based methods can keep up with silica membrane-based methods, while other publications assert that silica membrane-based methods are superior^20,21,30,46^. Nevertheless, research groups using the QIAamp Circulating Nucleic Acid kit (QIAGEN), a silica-based-method, consistently obtained ccfDNA yields higher than those attained via bead-based methods (for a detailed overview of the literature, see Supplemental Table S1)^20,61^. We conclude from these findings and our results that the choice of method for ccfDNA extraction (bead-based vs. silica membrane, automated vs. manual) should be based on the anticipated output, the level of throughput required, and the necessity or otherwise of automation. Fully automated, bead-based techniques will be crucial to high-throughput applications in routine clinical settings.

Interestingly, the correlation of the ccfDNA yields from the EZ2 and Maxwell extraction methods differed depending on the quantification platforms used. On all quantification platforms used in our research, the angle of the correlation line was lower than the bisector, confirming generally higher ccfDNA yields from use of the EZ2 instrument. The correlation curve of the qPCR data was closer to the bisector line than the correlation curves of the fluorescence quantification and the electrophoresis data. Therefore, while there may be higher concentrations of ccfDNA in EZ2 eluates, the effect is smaller when looking at qPCR data, a method that measures the amplifiability of DNA templates. A low ratio of long to short fragments can reduce the amplifiability of a template, as the proportion of amplifiable fragments decreases when the fragments are too short^62^. Applied to our data, this could point toward differences in fragment size distribution between the Maxwell and the EZ2 eluates, resulting in differences in amplifiability.

We also compared the two extraction instruments for differences in fragment length distribution within the extracts. This is a well-documented quality control procedure that is crucial to cancer diagnosis and the predicting progression and prediction of prognosis^7,22,63^. Prior work has shown that the method of extraction used may affect fragment sizes within the samples^29,44^. Quantification of the copy numbers of ALU60 and ALU247 templates revealed differences in the distribution of fragment lengths between the isolates. We found that the numbers of ALU60 copies, representing all fragments longer than 60 bp, were significantly higher for EZ2 kit extracts than for those obtained with the Maxwell kit. Conversely, higher copy numbers of large fragments (ALU247) were extracted with the Maxwell instrument. Looking at the ratio of longer to-total fragment size, we note that ccfDNA integrity of plasma samples isolated by the Maxwell kit was significantly higher than that of EZ2 samples. In summary, it appears that the EZ2 instrument is superior in extracting total ccfDNA and small DNA fragments in particular. Research has found that high yields of short, fragmented DNA are more likely to contain higher percentages of ctDNA than lower yields and can therefore serve as a measure of quality^64^. Further, fragment size distribution is a characteristic relevant to the choice of a downstream application. For example, sequencing methods relying on double-stranded library preparation were shown to be insensitive on ultra-short fragments and should be rejected in favor of single-stranded library preparation to avoid depletion of short DNA molecules^65^.

Our results regarding integrity are in line with other studies showing integrities of between 0.5 and 0.8^7–9,18^. Notwithstanding differences in target design, most of the groups that conducted these studies determined integrity using an ALU-specific qPCR. However, the field remains without a standardized procedure for sample handling, ccfDNA isolation and determination of integrity^66^. These factors contribute to the high divergences in integrity values observed among different studies.

Differences between the isolation methods evaluated were particularly apparent after quantification of mtDNA. Median hmito copy numbers were 15.9-fold higher in the EZ2 samples than in the Maxwell probes. The literature observes that cell-free mtDNA of the plasma is highly enriched in short sizes (30–60 bp)^67^. This supports the assumption that the EZ2 kit is better able to extract short fragments than the Maxwell kit. While *ALU-*specific qPCR enables quantification of the major part of nuclear ccfDNA, it does not include the representative amount of circulating mtDNA levels. Many current studies are limited to nuclear ccfDNA quantification^20,30,68^. To our knowledge, this is the first report comparing different extraction methods with regard to specific yields of mtDNA. Current work has discussed circulating mtDNA as a non-invasive biomarker in several cancers^69,70^. Analysis of hmito copy numbers in ccfDNA enables specific quantification of mtDNA plasma levels^50,71^. High mtDNA yield is crucial to downstream analysis such as mutational screening; however, we are yet to completely understand the complex role of changes in mtDNA levels themselves and in mitochondria-specific alterations during cancer evolution and progression. Further research is therefore vital if we are to improve the use of mtDNA as a biomarker. Currently, the major challenge of working with mtDNA is its low concentration in plasma samples, which indicates the importance of techniques that enable the extraction of as much mtDNA as possible^67^.

The present study has some limitations. First, due to the small size of the study cohort, we did not compare values obtained from samples of breast cancer patients with those from healthy individuals. We are therefore unable to draw conclusions regarding the diagnostic potential of ccfDNA extracts. A larger cohort would be required in order to establish whether the method used for ccfDNA isolation impacts the sensitivity and specificity of ccfDNA yield and integrity for the purpose of distinguishing between healthy individuals and patients with breast cancer. Future studies might usefully investigate this question with the aim of providing more information on the diagnostic application of ccfDNA^9,27^. Second, the study focused solely on ccfDNA quantity and quality, but not on ctDNA detection. We did not perform any downstream analysis identifying mutant alleles and were therefore unable to estimate ctDNA yield. Further, we used slightly different plasma input conditions for the two isolation platforms; for the Maxwell kit, 1 ml of undiluted plasma was used, while for the EZ2 kit, 1 ml plasma was diluted with 1 ml 1xPBS. This adaptation was necessary and recommended by the manufacturer for the purpose of increasing the input volume to the recommenended 2 ml of plasma. However, such a dilution has the potential to influence the ccfDNA extraction process due to dilution of inhibitory substances present in the plasma sample.

Overall, this study presents novel data on the quantitative and time-related efficiency of two fully automated ccfDNA extraction instruments, EZ2 and Maxwell. Of these, the EZ2 instrument provided better results with respect to ccfDNA and mtDNA yield, while the Maxwell instrument appeared superior in isolating long fragments. We also confirmed that the method of isolation used has a direct effect on ccfDNA integrity, a factor which should be taken into account when comparing studies. This finding further emphasizes the need for standardized procedures and quality controls where ccfDNA analysis is used in a diagnostic or prognostic clinical setting. Supplementary quality control should additionally take place before downstream analysis; this should include evaluation of fragment size distribution, checking for contamination with DNA from white blood cells leading to the presence of clonal hematopoietic variants, and assessment of the purity of extracted ccfDNA (e.g. presence of PCR inhibitors such as heparin)^64,72^.

## Supplementary Information

Supplementary Information.

## Data Availability

The datasets generated and/or analyzed during the present study are available from the corresponding author on reasonable request.
