# Supplementary material for: Evaluation of automated techniques for extraction of circulating cell-free DNA for implementation in standardized high-throughput workflows
Source: Sci Rep. 2023 Jan 7;13:373. doi: 10.1038/s41598-022-27216-5 (PMC9825368; doi:10.1038/s41598-022-27216-5)
Supplement: Supplementary file 1 — Supplementary Information. [file 41598_2022_27216_MOESM1_ESM.docx]

**Supplemental Figure S1**


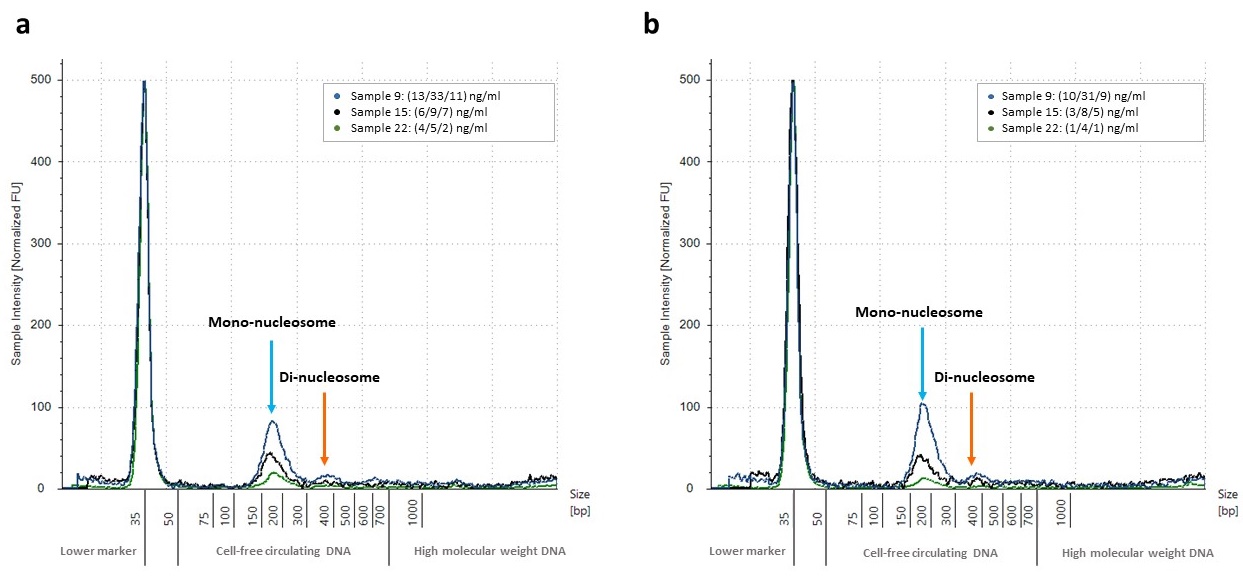


**Figure S1** DNA electrophoretic profiles from three patients, representing low-, medium- and high-concentration samples. DNA peak at 35 base pairs (bp) is a molecular-weight size marker. Fragments with a size above 700 bp are considered high molecular weight DNA. Peaks corresponding to the length of a single nucleosome (mononucleosome) are indicated by a blue arrow, while the orange arrows mark the peaks corresponding to the fragment size of two nucleosomes (dinucleosome). The sample concentration is given in brackets for all quantification methods in ng/ml plasma (Quantus fluorometer/ quantitative real-time-polymerase chain reaction / TapeStation). The DNA electrophoretic profiles are shown for the samples 9,15, and 22, isolated using (a) the EZ2 ccfDNA field test kit or (b) the Maxwell RSC ccfDNA plasma kit. The samples isolated using the EZ2 ccfDNA field test kit are in a final recovered volume of 65 µl elution buffer, and the samples isolated using the Maxwell RSC ccfDNA plasma kit are in a final recovered volume of 45 µl buffer.

**Supplementary Table S1** Overview of literature comparing ccfDNA isolation methods

| **Isolation method** | | **Quantification method** | **Results**  **Concentration of ccfDNA** | | **Reference** |
| --- | --- | --- | --- | --- | --- |
| **silica membrane** | **magnetic beads** |  | **silica membrane vs magnetic beads** | **QIAamp Circulating nucleic acid kit vs Maxwell RSC ccfDNA plasma kit** |  |
| QIAamp circulating nucleic acid (QIAGEN) | MagNA Pure Compact Nucleic Acid Isolation Kit I (Roche) Maxwell RSC ccfDNA plasma kit (Promega) | Qubit fluorometer (Thermo Fisher Scientific) Agilent Bioanalyzer (Agilent) ctDNA by ddPCR | equal | equal | (Pérez-Barrios et al. 2016) |
| QIAamp circulating nucleic acid kit (QIAGEN) | Maxwell RSC ccfDNA plasma kit (Promega) EpiQuick Circulating Cell-Free DNA Isolation Kit (EpiGentek) NEXTprep-Mag cfDNA Isolation Kit (SanBio) | Qubit fluorometer (Thermo Fisher Scientific) ddPCR | equal | equal | (Sorber et al. 2017) |
| QIAamp circulating nucleic acid kit (QIAGEN) | QIAsymphony circulating DNA kit (QIAGEN) | Qubit fluorometer (Thermo Fisher Scientific) qPCR | **magnetic beads >** silica membrane |  | (Wolf et al. 2016) |
| QIAamp circulating nucleic acid kit (QIAGEN) | Maxwell RSC ccfDNA plasma kit (Promega) QIAamp minElute ccfDNA mini kit (QIAGEN) MagMAX cell-free DNA isolation kit (Applied Biosystems) NextPrep-Mag cfDNA isolation kit (PerkinElmer) | BioRad Experion ddPCR analysis | **silica membrane >** magnetic beads | **QIAamp circulating nucleic acid kit >** Maxwell RSC ccfDNA plasma kit | (Diefenbach et al. 2018) |
| QIAamp circulating nucleic acid kit (QIAGEN) | Maxwell RSC ccfDNA plasma kit (Promega) QiAsymphony DSP circulating DNA kit (QIAGEN) | methylation-specific qPCR | equal |  | (Deger et al. 2021) |
|  | Maxwell RSC ccfDNA plasma kit (Maxwell) QiAamp minElute ccfDNA mini kit (QIAGEN) | qPCR Quantus Fluorometer (Promega) |  |  | (Huebner et al. 2021) |
| QIAamp circulating nucleic acid kit (QIAGEN) Avenio cfDNA isolation kit (Roche) Cobas cfDNA SP kit (Roche) | MagMAX Cell-Free DNA Isolation Kit (Applied Biosystems) MinElute (QIAGEN) QiaSymphony robot with DSP circulating DNA kit (QIAGEN) | Qubit fluorometer (Thermo Fisher Scientific) BioAnalyzer chips (Agilent) TapeStation (Agilent) | equal |  | (Koessler et al. 2020) |
| QIAamp circulating nucleic acid kit (QIAGEN) Zymo Quick ccfDNA Serum & Plasma Kit (Zymo Research) | Maxwell RSC ccfDNA plasma kit (Promega) QIAamp MinElute ccfDNA midi kit (QIAGEN) | Qubit fluorometer (Thermo Fisher Scientific) qPCR | equal | **QIAamp circulating nucleic acid kit >** Maxwell RSC ccfDNA plasma kit | (Leest et al. 2020) |
| QIAamp circulating nucleic acid kit (QIAGEN) | QIAsymphony circulating DNA kit (QIAGEN) QIAamp MinElute ccfDNA kit (QIAGEN) Maxwell RSC ccfDNA plasma kit AX1115 (Promega) Maxwell RSC ccfDNA plasma kit AS1480 (Promega) Chemagic CNA 4k kit special (PerkinElmer) | Qubit fluorometer (Thermo Fisher Scientific) qPCR Quantiplex Pro assay (QIAGEN) | equal | **QIAamp circulating nucleic acid kit >** Maxwell RSC ccfDNA plasma kit | (Lampignano et al. 2020) |
| QIAamp circulating nucleic acid kit (QIAGEN) | QIAsymphony DSP circulating DNA kit (QIAGEN) | qPCR | equal |  | (Stray and Zimmermann 2019) |
| QIAamp circulating nucleic acid kit (QIAGEN) NucleoSpin Plasma XS (Macherey-Nagel, Hoerdt, France) Plasma/Serum Circulating DNA Purification Mini Kit and Norgen Plasma/Serum Cell-Free Circulating DNA Purification Mini Kit (Norgen) | Chemagic NA Extraction Kit (PerkinElmer) | Qubit fluorometer (Thermo Fisher Scientific); qPCR | **silica membrane >** magnetic beads |  | (Mauger et al. 2015) |
| QIAamp circulating nucleic acids kit (QIAGEN) | Maxwell RSC ccfDNA plasma kit (Promega) QIAsymphony (QIAGEN) | qPCR Qubit fluorometer (Thermo Fisher Scientific) | equal | **QIAamp circulating nucleic acid kit >** Maxwell RSC ccfDNA plasma kit | (van Dessel et al. 2019) |
| QIAamp circulating nucleic acids kit (QIAGEN) | QIAamp DNA Blood Mini kit (QIAGEN) QIASymphony DSP Virus kit (QIAGEN) | qPCR | equal |  | (Warton et al. 2018) |
| High Pure PCR Template Preparation Kit (Roche) (glass fiber membrane column) | automated MagNA Pure LC Instrument (Roche Diagnostics)  using the LC DNA Isolation Kit | qPCR | equal |  | (Banzola et al. 2008) |
| QIAamp circulating nucleic acid kit (QIAGEN) | Maxwell RSC ccfDNA plasma kit (Promega) | Bioanalyzer (Agilent) Qubit fluorometer (Thermo Fisher Scientific) | **silica membrane >** magnetic beads | **QIAamp circulating nucleic acid kit >** Maxwell RSC ccfDNA plasma kit | (Kloten et al. 2017) |
| Plasma/Serum Cell-Free Circulating DNA Purification Kit (Norgen), Quick-cfDNA™ Serum and Plasma kit (Zymo Research) QIAamp circulating nucleic acid kit (QIAGEN) | Maxwell RCS ccfDNA plasma kit (Promega) NextPrep-Mag™ cfDNA Isolation kit (Bioo Scientific) | Qubit fluorometer (Thermo Fisher Scientific) TapeStation (Agilent) qPCR | equal | equal | (Solassol et al. 2018) |
| Virus combo kit 24C-LVX480-1000 (Taigen) | MagNA Pure 24 Total NA Isolation Kit (Roche) NextPrep-Mag cfDNA isolation kit (PerkinElmer) IDXTRACT-MAG kit (IDSolution) | ddPCR Qubit Fluorometer (Thermo Fisher Scientific) BIABooster | equal |  | (Pedini et al. 2021) |
| QIAamp circulating nucleic acid kit (QIAGEN) AmoyDxVR Circulating DNA kit (Amoy Diagnostics) MicrodiagVR circulating DNA isolation kit (MicroDiag Biomedicine) | MagMAX cell-free DNA isolation kit (Thermo Fisher Scientific) | Qubit Fluorometer (Thermo Fisher Scientific) Bioanalyzer (Agilent) | equal |  | (Wang et al. 2021) |

qPCR: quantitative polymerase chain reaction; ctDNA: circulating tumor DNA; ddPCR: droplet digital polymerase chain reaction

**References**

Banzola, I., I. Kaufmann, O. Lapaire, S. Hahn, W. Holzgreve, and C. Rusterholz. 2008. 'Isolation of serum nucleic acids for fetal DNA analysis: comparison of manual and automated extraction methods', *Prenat Diagn*, 28: 1227-31.

Deger, T., R. G. Boers, V. de Weerd, L. Angus, M. M. J. van der Put, J. B. Boers, Z. Azmani, IJcken W. F. J. van, D. J. Grünhagen, L. F. van Dessel, Mpjk Lolkema, C. Verhoef, S. Sleijfer, J. W. M. Martens, J. Gribnau, and S. M. Wilting. 2021. 'High-throughput and affordable genome-wide methylation profiling of circulating cell-free DNA by methylated DNA sequencing (MeD-seq) of LpnPI digested fragments', *Clin Epigenetics*, 13: 196.

Diefenbach, R. J., J. H. Lee, R. F. Kefford, and H. Rizos. 2018. 'Evaluation of commercial kits for purification of circulating free DNA', *Cancer Genet*, 228-229: 21-27.

Huebner, H., H. Lubrich, S. Blum, S. Antoniadis, J. Lermann, A. Ekici, P. A. Fasching, M. W. Beckmann, M. Ruebner, and S. Burghaus. 2021. 'Comparison of methods for isolation and quantification of circulating cell-free DNA from patients with endometriosis', *Reprod Biomed Online*, 43: 788-98.

Kloten, V., N. Rüchel, N. O. Brüchle, J. Gasthaus, N. Freudenmacher, F. Steib, J. Mijnes, J. Eschenbruch, M. Binnebösel, R. Knüchel, and E. Dahl. 2017. 'Liquid biopsy in colon cancer: comparison of different circulating DNA extraction systems following absolute quantification of KRAS mutations using Intplex allele-specific PCR', *Oncotarget*, 8: 86253-63.

Koessler, T., V. Paradiso, S. Piscuoglio, R. Nienhold, L. Ho, Y. Christinat, L. M. Terracciano, G. Cathomas, A. Wicki, T. A. McKee, and T. Nouspikel. 2020. 'Reliability of liquid biopsy analysis: an inter-laboratory comparison of circulating tumor DNA extraction and sequencing with different platforms', *Lab Invest*, 100: 1475-84.

Lampignano, R., M. H. D. Neumann, S. Weber, V. Kloten, A. Herdean, T. Voss, D. Groelz, A. Babayan, M. Tibbesma, M. Schlumpberger, F. Chemi, D. G. Rothwell, H. Wikman, J. P. Galizzi, I. Riise Bergheim, H. Russnes, B. Mussolin, S. Bonin, C. Voigt, H. Musa, P. Pinzani, E. Lianidou, G. Brady, M. R. Speicher, K. Pantel, F. Betsou, E. Schuuring, M. Kubista, W. Ammerlaan, M. Sprenger-Haussels, T. Schlange, and E. Heitzer. 2020. 'Multicenter Evaluation of Circulating Cell-Free DNA Extraction and Downstream Analyses for the Development of Standardized (Pre)analytical Work Flows', *Clin Chem*, 66: 149-60.

Leest, P. V., P. A. Boonstra, A. T. Elst, L. C. V. Kempen, M. Tibbesma, J. Koopmans, A. Miedema, M. Tamminga, H. J. M. Groen, A. K. L. Reyners, and E. Schuuring. 2020. 'Comparison of Circulating Cell-Free DNA Extraction Methods for Downstream Analysis in Cancer Patients', *Cancers (Basel)*, 12.

Mauger, F., C. Dulary, C. Daviaud, J. F. Deleuze, and J. Tost. 2015. 'Comprehensive evaluation of methods to isolate, quantify, and characterize circulating cell-free DNA from small volumes of plasma', *Anal Bioanal Chem*, 407: 6873-8.

Pedini, P., H. Graiet, L. Laget, L. Filosa, J. Chatron, N. Cherouat, J. Chiaroni, L. Hubert, C. Frassati, and C. Picard. 2021. 'Qualitative and quantitative comparison of cell-free DNA and cell-free fetal DNA isolation by four (semi-)automated extraction methods: impact in two clinical applications: chimerism quantification and noninvasive prenatal diagnosis', *J Transl Med*, 19: 15.

Pérez-Barrios, C., I. Nieto-Alcolado, M. Torrente, C. Jiménez-Sánchez, V. Calvo, L. Gutierrez-Sanz, M. Palka, E. Donoso-Navarro, M. Provencio, and A. Romero. 2016. 'Comparison of methods for circulating cell-free DNA isolation using blood from cancer patients: impact on biomarker testing', *Transl Lung Cancer Res*, 5: 665-72.

Solassol, J., X. Quantin, M. Larrieux, R. Senal, P. Audran, D. Grand, A. Mangé, E. P. Diamandis, and J. A. Vendrell. 2018. 'Comparison of five cell-free DNA isolation methods to detect the EGFR T790M mutation in plasma samples of patients with lung cancer', *Clin Chem Lab Med*, 56: e243-e46.

Sorber, L., K. Zwaenepoel, V. Deschoolmeester, G. Roeyen, F. Lardon, C. Rolfo, and P. Pauwels. 2017. 'A Comparison of Cell-Free DNA Isolation Kits: Isolation and Quantification of Cell-Free DNA in Plasma', *J Mol Diagn*, 19: 162-68.

Stray, J., and B. Zimmermann. 2019. 'Isolation of Cell-Free DNA from Maternal Plasma', *Methods Mol Biol*, 1885: 309-23.

van Dessel, L. F., S. R. Vitale, J. C. A. Helmijr, S. M. Wilting, M. van der Vlugt-Daane, E. Oomen-de Hoop, S. Sleijfer, J. W. M. Martens, Mphm Jansen, and M. P. Lolkema. 2019. 'High-throughput isolation of circulating tumor DNA: a comparison of automated platforms', *Mol Oncol*, 13: 392-402.

Wang, M., X. Huang, X. Li, Q. Guo, W. Xu, M. Zhao, X. Wang, L. Wang, and J. Lou. 2021. 'Performance comparison of commercial kits for isolating and detecting circulating tumor DNA', *Scand J Clin Lab Invest*, 81: 276-81.

Warton, K., L. J. Graham, N. Yuwono, and G. Samimi. 2018. 'Comparison of 4 commercial kits for the extraction of circulating DNA from plasma', *Cancer Genet*, 228-229: 143-50.

Wolf, A., K. Beller, S. Groemminger, W. Hofmann, M. Sachse, and J. Fassunke. 2016. 'Purification of Circulating Cell-Free DNA from Plasma and Urine Using the Automated Large-Volume Extraction on the QIAsymphony® SP Instrument', *Adv Exp Med Biol*, 924: 179-85.
